# An Innovative Patient Stratification Tool Integrating Clinical and Economic Data for Benchmarking Oncology and Hematology Care: The PATONCOS System

**DOI:** 10.3390/jcm15114374

**Published:** 2026-06-05

**Authors:** Raquel Moreno-Díaz, Alejandra Melgarejo-Ortuño, Beatriz Monje-García, Laura Delgado-Téllez de Cepeda, Ana Beatriz Fernández-Román, Marta Manso-Manrique, Javier Letéllez-Fernández, Beatriz Candel-García, Amelia Sánchez-Guerrero, Miguel Ángel Amor-García, Mario García-Gil, Maria Isabel Valverde-Merino, Francisco Javier García-Sánchez, Miguel Ángel Calleja-Hernández

**Affiliations:** 1 Pharmacy Service, Hospital Universitario Infanta Cristina, Instituto de Investigación Sanitaria Hospital Puerta de Hierro Segovia Arana (IDIPHISA), 28981 Parla, Spain; rmorenod@salud.madrid.org (R.M.-D.); alejandra.melgarejo@salud.madrid.org (A.M.-O.); miguelangel.amor@salud.madrid.org (M.Á.A.-G.); 2 Pharmacy Service, Hospital Universitario del Henares, 28822 Coslada, Spain; beatriz.monje@salud.madrid.org; 3 Pharmacy Service, Hospital Universitario Puerta de Hierro, Instituto de Investigación Sanitaria Hospital Puerta de Hierro Segovia Arana (IDIPHISA), 28222 Majadahonda, Spain; laura.delgado@salud.madrid.org (L.D.-T.d.C.); marta.manso@salud.madrid.org (M.M.-M.); asguerrero@salud.madrid.org (A.S.-G.); 4 Pharmacy Service, Hospital Universitario de Fuenlabrada, 28942 Fuenlabrada, Spain; afroman@salud.madrid.org (A.B.F.-R.); javier.letellez@salud.madrid.org (J.L.-F.); beatriz.candel@salud.madrid.org (B.C.-G.); mgarciagil@salud.madrid.org (M.G.-G.); 5 Pharmaceutical Care Research Group, University of Granada, 18010 Granada, Spain; misabelvalverdemerino@gmail.com; 6 Emergency Room Service, Surgical Prehabilitation Unit, Hospital Universitario Infanta Cristina, Instituto de Investigación Sanitaria Hospital Puerta de Hierro Segovia Arana (IDIPHISA), 28981 Madrid, Spain; 7 Medical Department, Faculty of Medicine, University Complutense of Madrid, 28040 Madrid, Spain; 8 Pharmacy Service, Hospital Universitario Virgen de las Nieves, 18014 Granada, Spain; mangel.calleja.sspa@juntadeandalucia.es

**Keywords:** patient stratification, oncology, hematology, healthcare costs, pharmacoeconomics, biomarkers, targeted therapy, cost analysis, resource allocation, real-world data, clinical decision-making, oncology pharmacotherapy, benchmarking, precision medicine, healthcare management

## Abstract

**Background:** The growing complexity and cost of oncohematological treatments has created an urgent need for standardized methodologies capable of enabling inter-institutional comparisons of healthcare expenditure within homogeneous patient groups. Cancer-related pharmaceutical costs vary substantially depending on tumour type, disease stage, and therapeutic approach, making cross-institutional benchmarking challenging due to heterogeneity in patient populations and clinical practice patterns. Therefore, integrating cost analysis with clinically meaningful patient stratification is essential to improve resource allocation and outcome evaluation. **Methods:** A multicentre working group comprising four tertiary hospitals in Madrid (Spain) was established to develop and preliminarily evaluate a novel classification system for adult oncohematological patients. A standardized methodology was designed to stratify patients into homogeneous groups (PATONCO categories) based on tumor location, therapeutic objective, and clinically relevant biomarkers. A cost indicator was defined as the average cost per patient per month for each PATONCO category. Data were extracted from pharmacy dispensing systems and analyzed using descriptive and inferential statistics, including Kruskal–Wallis and post hoc Dunn tests. **Results:** A total of 3659 patients were included (3168 oncology; 491 hematology), distributed across 62 programmes (54 oncology; 8 hematology). The PATONCOS tool enabled the identification and validation of a cost indicator (average cost/patient/month per category), allowing inter-hospital comparison. Significant differences in costs were observed across most high-prevalence categories, reflecting variability in drug selection within homogeneous patient groups, as documented by the differential use of specific therapeutic agents across centers. The model demonstrated its capacity to detect intra-group homogeneity and inter-group variability, improving the identification of high-cost patient subgroups and supporting benchmarking across centers. **Conclusions:** The PATONCOS tool provides a novel, clinically oriented stratification methodology that integrates pharmacotherapy, biomarkers, and disease stage with economic evaluation. This approach enables more accurate comparisons of oncology treatment costs between institutions and may support data-driven decision-making in resource allocation. Its implementation may contribute to more sustainable healthcare systems by aligning clinical practice with economic outcomes.

## 1. Introduction

Cancer remains one of the leading causes of morbidity and mortality worldwide. The International Agency for Research on Cancer estimated approximately 19.3 million new cases in 2020, with projections reaching 30.2 million annually by 2040 [1]. In parallel with this growing burden, rapid scientific progress in oncology has led to significant advances in diagnostic tools and the development of novel targeted therapies [2]. Many of these therapies have improved clinical outcomes and, in some cases, reduced toxicity profiles, although their economic impact remains insufficiently characterized [3,4,5,6,7,8].

Despite demonstrated efficacy in clinical trials, the real-world impact of new oncological therapies—particularly in economic terms—is rarely evaluated before market introduction, and post-marketing analyses are complex and heterogeneous [9,10]. Healthcare costs in oncology vary substantially depending on tumor type, disease stage, and time since diagnosis [11,12], with particularly high costs observed at diagnosis and throughout the patient’s lifetime [13,14]. However, due to the complexity of healthcare information systems and variability in clinical practice, it remains difficult to accurately quantify and compare cancer-related costs between institutions, even within the same geographical region [15].

To address this challenge, organizations such as the American Society of Clinical Oncology (ASCO) and the European Society for Medical Oncology (ESMO) have developed frameworks to evaluate the cost–benefit relationship of oncological therapies [16,17]. Nevertheless, these approaches are primarily treatment-centered and do not fully account for patient-level heterogeneity, limiting their applicability for comparing real-world outcomes across institutions.

Hospital pharmacy services are uniquely positioned to undertake this integration. As part of their core mission—the rational use of medicines—pharmacists perform the clinical validation of every oncological prescription, generating structured data automatically at each point of care: patient identification, PATONCO category, therapeutic regimen, dosage form, and cost. This makes pharmacy-derived data an efficient and reliable source for patient-level economic analysis without requiring additional registration systems. Hospital pharmacy services routinely manage large volumes of data related to pharmacological treatments and associated costs in oncohematological patients. Integrating this information with clinically meaningful patient stratification could facilitate both economic evaluation and outcome assessment. In this context, reducing clinical variability and standardizing therapeutic approaches is essential to ensure the efficient and sustainable use of healthcare resources [11]. Therefore, there is an urgent need to develop methodologies that combine patient stratification with cost analysis to support long-term planning and benchmarking. Existing approaches—including cost analyses stratified by tumour type or treatment phase—often include heterogeneous patient populations, non-standardized cost components, or static classification criteria that cannot adapt to disease progression or changes in therapeutic strategy. These limitations prevent meaningful and reproducible inter-institutional comparisons [4,7,12,18].

Classification criteria play a critical role in defining homogeneous patient cohorts and ensuring comparability between studies [19]. Current cancer classification systems are primarily based on histopathological criteria, such as the International Classification of Diseases for Oncology (ICD-O), which focuses on disease coding but does not adequately capture clinical or economic heterogeneity [2,7,10,11]. More advanced classifications, such as the WHO Blue Books and the American Joint Committee on Cancer (AJCC) staging system, incorporate molecular features and biomarkers, highlighting their increasing relevance in oncology [20]. However, these systems remain insufficient for stratifying patients according to therapeutic approaches and associated costs.

The development of a clinically and economically meaningful classification system for oncology patients represents both a significant challenge and an opportunity for healthcare systems.

In this context, the aim of the PATONCOS tool is to establish a novel working methodology based on the stratification of homogeneous oncohematological patient groups, integrating clinical characteristics and therapeutic determinants in order to enable cost comparisons and evaluations of therapeutic approaches across medical centers, ultimately identifying opportunities for improvement in clinical practice and resource allocation.

## 2. Materials and Methods

### 2.1. Study Design and Settings

A retrospective multicenter observational study was conducted through a collaborative working group composed of hospital pharmacists specialized in oncology from four tertiary hospitals in Madrid (Spain): Puerta de Hierro University Hospital, Fuenlabrada University Hospital, Infanta Cristina University Hospital, and Henares University Hospital. It should be acknowledged that participating centers were selected based on shared regional context, comparable pharmacy software, and similar clinical workflows. While this approach enhances internal standardization, it may limit the generalizability of the findings to institutions with different healthcare infrastructures or prescribing environments.

**Figure 1:**
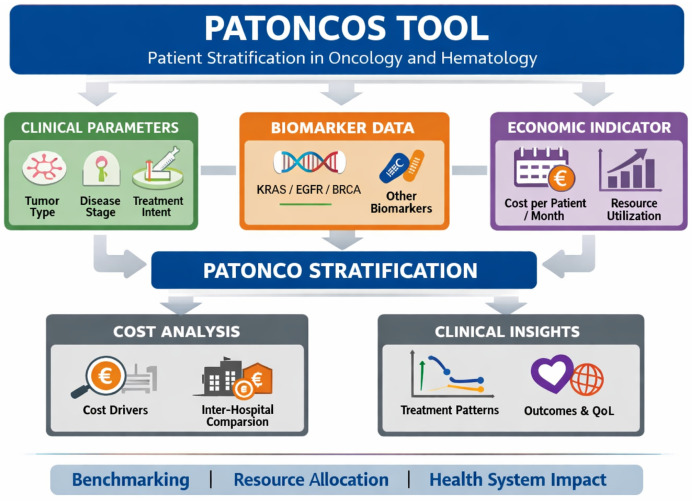
Conceptual framework of the PATONCOS tool. The model integrates clinical parameters (tumour type, disease stage, and treatment intent), biomarker data, and economic indicators to stratify patients into homogeneous groups (PATONCO categories). This stratification enables cost analysis and clinical insights, supporting benchmarking and resource allocation across healthcare systems.

The combined catchment area covered a population of over 1,085,000 inhabitants. The working group was established in December 2018, with monthly meetings held to develop, evaluate, and refine the methodology. The tool was named PATONCOS as an acronym for PATient ONCOlogy Stratification (Figure 1).

The study protocol was submitted to the Research Ethics Committee of the Puerta de Hierro Hospital Foundation (Majadahonda, Madrid) for registration and validation.

### 2.2. Development of the PATONCO Classification System

A standardized work procedure was designed to unify the classification of adult oncohematological patients across participating centers. The classification system was reviewed through multidisciplinary collaboration with oncologists and hematologists and hospital pharmacists from all participating centers. Validation was conducted through formal consensus meetings in which each PATONCO category was reviewed, discussed, and approved. Inter-rater discrepancies in category assignment were resolved through iterative discussion until full consensus was reached. An initial pilot phase, conducted between May and September 2019, allowed for the identification and correction of classification errors prior to full implementation. Although structured multidisciplinary consensus meetings were conducted throughout the development process, no formal Delphi methodology or inter-rater reliability analysis was performed. This represents a limitation of the current validation framework that future studies should address.

The classification system (PATONCO categories) was developed based on variables that determine therapeutic decision-making, including tumor location, therapeutic objective (adjuvant, neoadjuvant, or metastatic), and the presence of clinically relevant biomarkers.

Selected biomarkers included ALK, EGFR, BRCA, PIK3CA, KRAS, NRAS, HER2, and hormone receptors (HR). Biomarkers without a direct impact on therapeutic decision-making, such as Ki-67, were excluded. In hematological malignancies, clinical conditions influencing treatment strategies—such as eligibility for hematopoietic transplantation—were also incorporated.

Baseline characteristics for each cancer type, including diagnostic criteria, pathological features, molecular alterations, and treatment options, were defined to ensure standardization. The classification system was validated through multidisciplinary collaboration with oncologists and hematologists.

### 2.3. Implementation and Data Collection

The PATONCO classification was integrated into the pharmacy prescription software of each participating center, enabling the automatic classification of all oncohematological patients with electronic prescriptions. This allowed systematic data extraction and inter-center comparability.

The implementation phase took place between May and September 2019, during which discrepancies and potential errors were identified and resolved through working group meetings. Patients enrolled in clinical trials were excluded to avoid potential bias.

A structured database was developed as part of a big data architecture. Monthly data extraction included patient identification, PATONCO category, treatment, dosage form, unit dose, and cost. The database was updated continuously and closed annually to allow for longitudinal comparisons. Data quality was ensured through systematic cross-validation between centers during monthly working group meetings. Missing or inconsistent records were identified and resolved through direct verification with the responsible pharmacy units at each site. A standardized data dictionary was applied uniformly across all participating hospitals to support interoperability between their respective pharmacy information systems.

**Figure 2:**
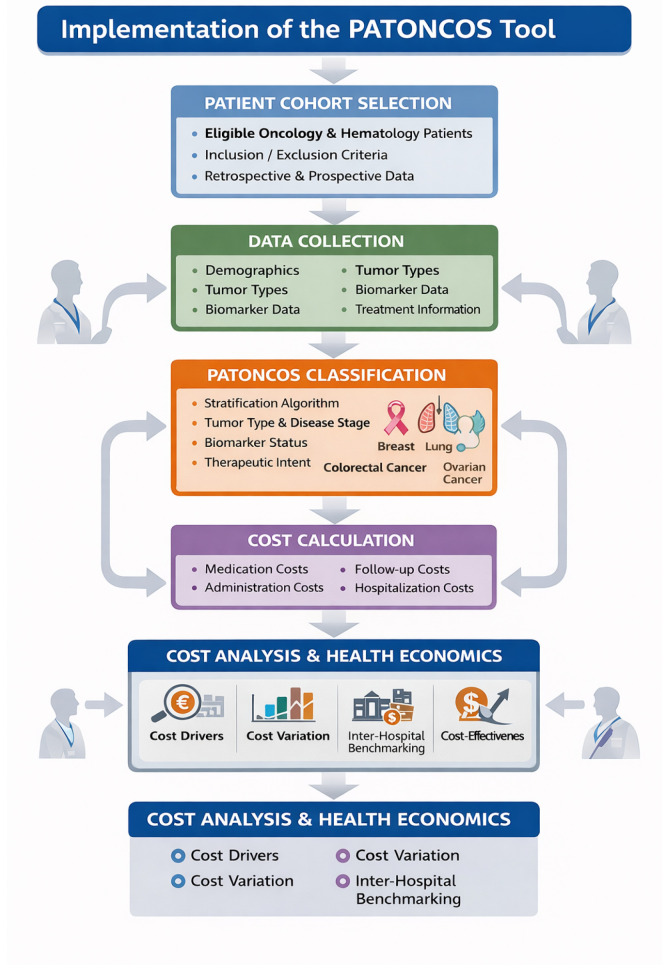
Workflow of the PATONCOS tool implementation. The process includes patient cohort selection, data collection, classification into PATONCO categories based on clinical and biomarker variables, cost calculation, and subsequent health economic analysis. This structured approach enables identification of cost drivers, inter-hospital variability, and benchmarking across centres.

The operational workflow of the PATONCOS tool, including patient selection, data collection, classification, cost calculation, and subsequent economic analysis, is illustrated in Figure 2.

### 2.4. Cost Definition and Economic Indicator

Cost analysis was based exclusively on direct pharmaceutical costs corresponding to marketed medicinal products, according to the Spanish Agency for Medicines and Health Products. Supportive therapies, indirect costs, and intangible costs were excluded.

A key economic indicator was defined as the average cost per patient per month for each PATONCO category. This indicator was calculated as the mean monthly cost of pharmacological treatment per patient within each category. Calculations were standardized using Microsoft Access, including the estimation of patient numbers and standard deviation.

For benchmarking purposes, procurement prices were monitored at 6, 12, and 18 months to minimize inter-hospital variability.

### 2.5. Statistical Analysis

Statistical analysis was performed using IBM SPSS Statistics version 25. Normality was assessed using the Kolmogorov–Smirnov test. No missing data were identified, as all records were extracted directly from pharmacy dispensing systems with mandatory fields. No formal sensitivity or outlier analyses were conducted beyond the exclusion of clinical trial patients. Clustering by hospital was implicitly addressed through the stratified analytical design, with all comparisons performed at the category level across the four centres independently.

A descriptive analysis of monthly cumulative consumption was conducted for each hospital and PATONCO category, including measures of central tendency (mean and median) and measures of dispersion (standard deviation, minimum, and maximum), as well as normality testing.

Comparisons of average cost per patient per month across the four hospitals were performed using the Kruskal–Wallis test, given the non-normal distribution of variables. When statistically significant differences were identified, post hoc pairwise comparisons were conducted using Dunn’s test with Holm correction for multiple testing.

Statistical significance was set at *p* < 0.05, with high statistical significance defined as *p* < 0.01.

### 2.6. Study Period and Benchmarking

Once the classification system and methodology were fully established, economic benchmarking was performed using data collected in 2022. The comparison system remained operational throughout the study period, including during the COVID-19 pandemic.

Benchmarking was conducted using a cost-indicator-based approach, comparing the average cost per patient per month within each PATONCO category across the four institutions. To minimize inter-hospital price variability attributable to differential procurement, acquisition prices were harmonized at 6-, 12-, and 18-month intervals. Only PATONCO categories with more than 50 patients were included in the comparative analysis to ensure statistical robustness and clinical representativeness. This threshold was selected pragmatically to improve statistical stability; however, no formal power calculation was performed to derive it. At the time of analysis, the hematology component was partially developed, including multiple myeloma, Hodgkin lymphoma, and non-Hodgkin lymphoma.

## 3. Results

### 3.1. PATONCOS Tool Development

The main outcome of this multicenter project was the development of the PATONCOS tool, a structured working instrument based on a novel classification system for oncohematological patients. This tool enables the differentiation of patients according to therapeutic approaches and associated economic impacts.

The PATONCOS tool was designed as a structured working instrument comprising three integrated components: (1) a collaborative working methodology based on periodic multidisciplinary meetings and structured consensus processes across participating centres; (2) a standardized oncohematological patient classification system (PATONCO categories), enabling the definition of homogeneous patient groups; and (3) a cost indicator—defined as the average cost per patient per month within each PATONCO category—that enables reproducible inter-hospital economic comparison. The primary objective of the tool was the identification and preliminary evaluation of this cost indicator, to facilitate the comparison of clinical and economic variability across centres. The development of the tool was supported by a collaborative working group and implemented through a standardized methodology integrated into routine clinical practice.

**Figure 3:**
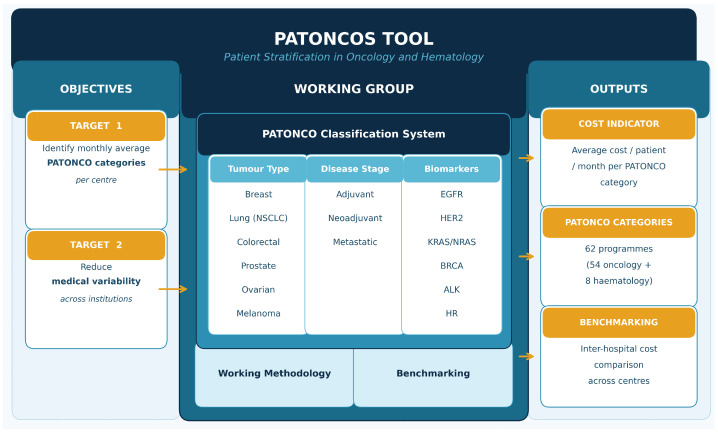
Elements of PATONCOS tool.

**Figure 4:**
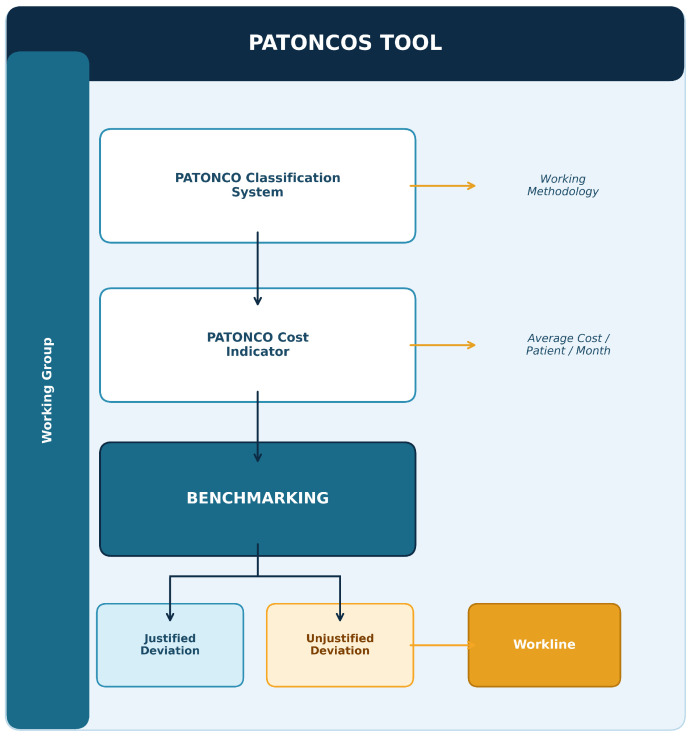
Working methodology flow.

This methodology allows for continuous benchmarking by generating comparative economic data between centers based on homogeneous patient groups. The workflow and structural components of the PATONCOS tool are illustrated in Figure 3 and Figure 4.

### 3.2. PATONCO Classification System

The PATONCOS classification system was defined based on tumor location, therapeutic objectives (adjuvant, neoadjuvant, or metastatic), and the presence of clinically relevant biomarkers that influence treatment decisions. Selected biomarkers included EGFR, HER2, NRAS, KRAS, BRCA, ALK, PIK3CA mutations, and hormone receptors.

Additional clinical conditions affecting therapeutic strategies were incorporated, particularly in hematological patients (e.g., eligibility for hematopoietic transplantation). A standardized nomenclature was established to ensure consistency and facilitate systematic analysis.

A total of 62 programs were identified according to diagnostic frequency and economic impact, including 54 oncology and 8 hematology categories (A representative subset of PATONCO categories is shown in Table 1 to illustrate the classification framework. The complete list of categories is provided in Supplementary Table S1). The classification system was designed as a dynamic structure, allowing updates according to the introduction of new therapies, biomarkers, and treatment indications.

### 3.3. Study Population

A total of 3659 patients were included in the analysis: 3168 in oncology categories and 491 in hematology categories. Among these, 2846 patients belonged to categories with more than 50 patients and were included in detailed comparative analyzes (2436 oncology; 410 hematology).

The most prevalent PATONCOS categories (defined a priori as those including more than 50 patients, consistent with the analytical threshold described in Section 3.3) were:

- Metastatic breast cancer HER2(−) HR(+)
- Metastatic non-squamous non-small cell lung cancer ALK(−) EGFR(−)
- Adjuvant colon cancer
- Metastatic colorectal cancer KRAS/NRAS mutated
- Metastatic colorectal cancer KRAS/NRAS wild-type
- Castration-resistant metastatic prostate cancer
- Multiple myeloma transplant candidate

Each of these categories included between 153 and 285 patients, representing the groups with the highest clinical and economic impact.

### 3.4. Cost Indicator and Inter-Hospital Comparison

The PATONCOS tool enabled the calculation of the average cost per patient per month for each PATONCO category, allowing direct comparison between hospitals.

Significant differences in cost were observed across most high-prevalence categories. Among the 25 categories with more than 50 patients, only two did not show statistically significant differences between hospitals. These cost differences were associated with variability in drug selection within homogeneous patient groups. As detailed in Section 3.5, specific therapeutic agents were used differentially across hospitals within the same PATONCO categories. Given that procurement prices were harmonized across centres, the observed cost variability is primarily attributable to differences in therapeutic strategies and the differential adoption of innovative treatments rather than to price differences.

The comparative analysis for 2022 (Table 2) includes the average cost per patient per month for each hospital and PATONCO category, as well as the mean deviation between centers. Higher patient volumes were observed in Hospital 1 and Hospital 3 compared to Hospitals 2 and 4.

### 3.5. Drivers of Cost Variability

Differences in cost between hospitals were primarily associated with variability in pharmacological treatment selection within the same PATONCO categories.

In metastatic breast cancer HER2(−) HR(+), variability was related to the use of everolimus, alpelisib, and the differential distribution of cyclin-dependent kinase inhibitors (ribociclib, abemaciclib, palbociclib).

In metastatic non-small cell lung cancer, the differences were mainly associated with the use of immunotherapies such as ipilimumab, nivolumab, and atezolizumab.

In the adjuvant colon cancer category, cost variability was associated with the use of pembrolizumab in MSI-H/dMMR stage III patients, as well as regorafenib and trifluridine/tipiracil in cases of exceptional or compassionate use. These agents are primarily indicated in metastatic settings; their presence in this category reflects real-world prescribing patterns and off-label use documented in pharmacy dispensing records, rather than standard adjuvant treatment.

In metastatic colorectal cancer, cost differences were driven by the use of trifluridine/tipiracil, aflibercept, and differences in prescribing anti-EGFR therapies such as panitumumab and cetuximab.

In castration-resistant metastatic prostate cancer, differences were associated with access to olaparib, enzalutamide, and the uptake of generic drugs.

In multiple myeloma transplant candidates, variability was related to the use of carfilzomib and access to daratumumab as first-line therapy.

These findings highlight that differences in therapeutic choices within homogeneous patient groups translate into measurable economic variability.

### 3.6. Budget Impact Distribution

Beyond individual cost indicators, the PATONCOS tool also enabled the analysis of the distribution of total healthcare expenditure by combining the average cost per patient per month with the number of patients in each category. The budget impact model highlights the substantial economic weight of high-prevalence categories, particularly metastatic lung and breast cancer, reinforcing the relevance of patient stratification for resource allocation. (Table 3).

**Figure 5:**
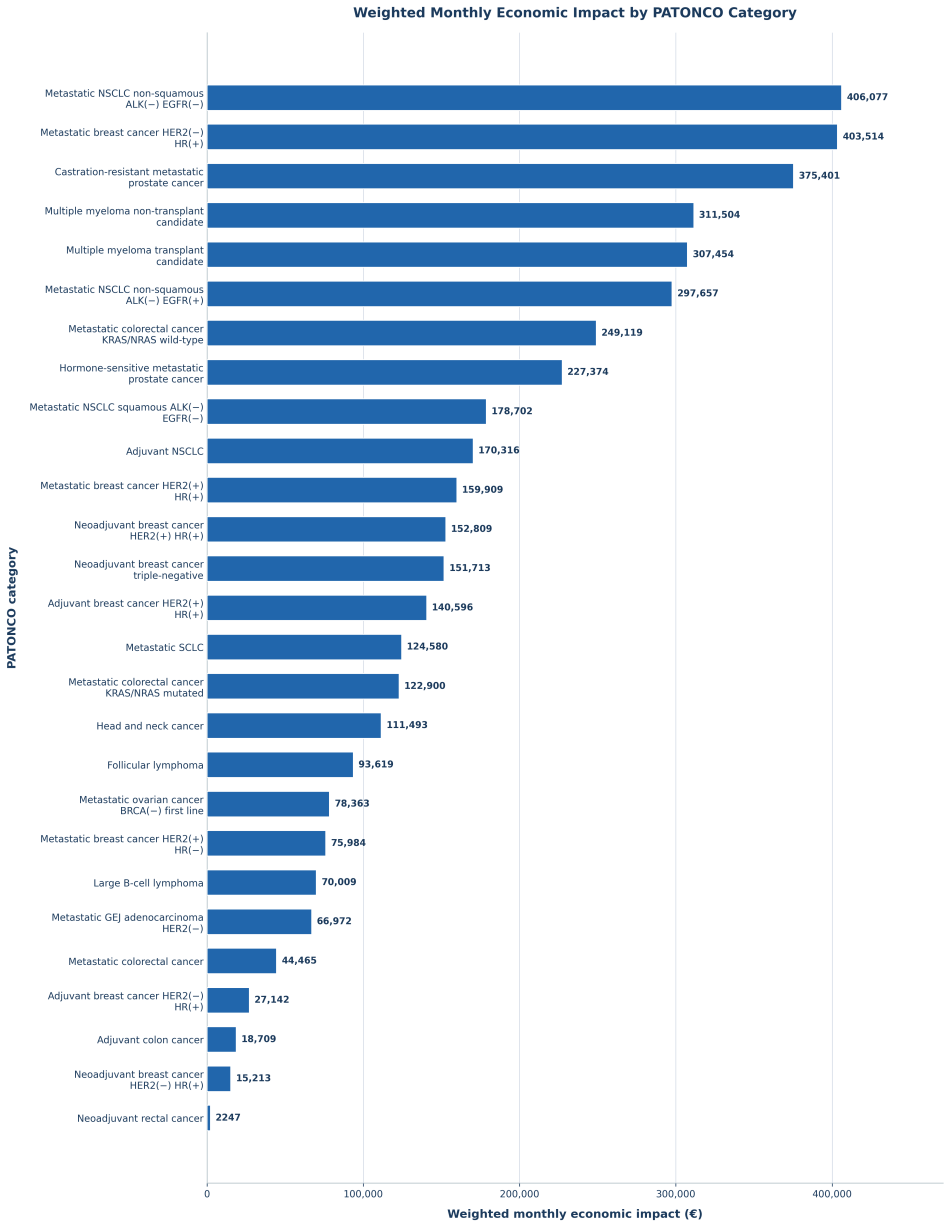
Weighted monthly economic impact by PATONCO category. The figure shows the relative budget burden of each category based on the average monthly cost per patient and the number of patients included in each group.

This approach provides a more realistic representation of budget impact, reflecting both treatment costs and patient volumes, and supporting more accurate financial planning and resource allocation. The weighted monthly economic impact by PATONCO category is illustrated in Figure 5, highlighting the categories with the greatest contribution to the overall budget burden.

## 4. Discussion

Based on a comprehensive review of the available literature, to our knowledge, few comparable methodologies have been identified that simultaneously integrates tumour location, disease stage, clinically relevant biomarkers, and an economic indicator to enable inter-centre comparison at the patient-group level. This study presents such an approach, though further literature review and external validation are needed to fully characterize its novelty within the broader landscape of oncology health economics. This approach provides a novel framework for understanding the economic impact of cancer treatment in real-world clinical practice.

The distribution of tumor types in our study is consistent with previously reported epidemiological patterns, with breast, colorectal, prostate, and lung cancers being the most prevalent [4,21,22,23,24,25]. Similarly, the distribution of patients across categories aligns with prior real-world data [4,26], supporting the external validity of the classification system.

The PATONCOS tool allows for the analysis of economic resource allocation among patients receiving different therapeutic strategies within homogeneous groups defined by tumor characteristics and biomarkers. Unlike traditional classification systems, this methodology enables dynamic patient stratification according to disease progression and therapeutic changes. The continuous updating of pharmacy dispensing data provides a real-time mechanism for identifying shifts in treatment patterns and associated costs.

This adaptability is particularly relevant in the current context of rapid therapeutic innovation in oncology and hematology. The system is designed to evolve in response to new drugs, emerging biomarkers, and changing treatment indications, ensuring that classification remains clinically meaningful and economically relevant over time.

Existing frameworks, such as the ESMO-Magnitude of Clinical Benefit Scale (ESMO-MCBS), focus primarily on evaluating the clinical benefit of therapies rather than patient-level stratification [27]. While valuable, these approaches do not allow direct comparison of similar patient groups across institutions, limiting their utility for benchmarking. Other economic studies have analyzed costs by tumor type or treatment phase, but they often include heterogeneous patient populations or incorporate non-pharmacological costs, making comparisons difficult [28,29].

The rapid acceptance and implementation of the PATONCO classification by oncology and hematology teams across participating centers highlights its feasibility and clinical applicability. Its integration into routine clinical workflows demonstrates that it can be adopted without significant disruption, even in complex healthcare environments. Notably, the tool remained operational during the COVID-19 pandemic, indicating robustness and low dependency on additional resources.

One of the main strengths of this approach is its ability to identify variability in pharmacological treatment within homogeneous patient groups. However, the high standard deviations observed in several PATONCO categories—most notably in adjuvant colon cancer (SD up to 568.66 in H2) and multiple myeloma transplant candidates (where SD values exceeded means in some hospitals)—indicate that substantial within-group economic heterogeneity persists. This suggests that, despite the clinical rationale underlying the classification criteria, therapeutic variability within categories remains considerable. Additional stratification variables—such as treatment line, performance status, or specific molecular subtype—may be necessary in future iterations to improve within-group homogeneity.Differences in drug selection, access to innovative therapies, and prescribing patterns were reflected in measurable cost differences between hospitals. This variability may be partially influenced by hospital volume, although our data do not allow for definitive conclusions in this regard [11].

Previous studies have typically evaluated the annual cost per patient based on aggregated expenditure divided by the total number of treated patients, without accounting for clinical heterogeneity [4]. In contrast, the PATONCOS methodology introduces a more granular approach by analyzing the cost per patient per month within clinically defined subgroups. This allows for a more precise identification of cost drivers, particularly in the context of targeted therapies, immunotherapy, and novel hormonal agents.

The increasing economic burden of oncology treatments is well documented and is largely driven by the introduction of targeted therapies, including anti-HER2 and anti-EGFR agents, tyrosine kinase inhibitor, mTOR inhibitors, and cyclin-dependent kinase inhibitors [4,23]. Our findings are consistent with these observations and further demonstrate how differences in the adoption of these therapies contribute to inter-hospital variability.

In prostate cancer and multiple myeloma, similar trends have been reported, with significant cost increases associated with new therapeutic agents such as enzalutamide, abiraterone, carfilzomib, and daratumumab [12,14,30]. However, previous analyzes often included broader healthcare costs or different methodological approaches, limiting direct comparison with our results.

The PATONCOS tool also enables the evaluation of budget impact by combining cost per patient per month with patient volume, providing a more realistic representation of healthcare expenditure. This dual perspective is essential for decision-making processes related to resource allocation and planning. The implementation workflow (Figure 2) enabled systematic and reproducible data collection across centers.

Despite its strengths, this study has several limitations that should be acknowledged. First, this analysis does not incorporate clinical outcome data—such as overall survival, progression-free survival, or quality of life—which precludes conclusions about value-based care or the relationship between cost and therapeutic effectiveness. This is a recognized limitation that future research should address by linking PATONCO categories to longitudinal outcome registries. Second, potential confounding factors—including disease severity, patient comorbidity burden, treatment sequencing, and institutional referral patterns—were not systematically captured and may contribute to the observed inter-hospital cost variability independently of drug selection. Third, participating centres were chosen based on shared regional context and comparable pharmacy systems, which may limit generalizability to institutions with different healthcare infrastructures or clinical workflows. Fourth, the PATONCOS classification requires continuous updating to reflect ongoing therapeutic innovation. Fifth, the haematology component remains partially developed, and further work is needed to expand its scope to additional malignancies.

Future research should focus on integrating clinical outcomes, such as survival and quality of life, with economic indicators. This would allow for a more comprehensive assessment of value in oncology care and further strengthen the role of tools such as PATONCOS in healthcare decision-making.

From a health policy perspective, the PATONCOS tool provides actionable data to support formulary management, budget impact modelling, and regional healthcare planning. By identifying cost drivers within homogeneous patient groups, hospital pharmacy committees and clinical directors can prioritize areas for pharmacoeconomic review and engage more effectively with payers and health authorities. Moreover, unjustified inter-hospital cost variability detected through benchmarking may reflect inequities in access to innovative therapies, a hypothesis that warrants dedicated investigation from a health equity standpoint. Overall, this study supports the need for new methodologies that align clinical stratification with economic evaluation. By enabling real-time, patient-centered cost analysis, the PATONCOS tool provides a practical framework for improving transparency, reducing variability, and promoting more efficient and sustainable use of healthcare resources.

## 5. Conclusions

This study presents a novel approach for the classification of oncohematological patients that integrates clinical characteristics, therapeutic determinants, and economic evaluation within a unified framework. It should be noted that the present study evaluates pharmaceutical cost variability only. Clinical outcomes—including survival, toxicity, quality of life, and value-based care metrics—were not assessed. Cost variability between institutions should not be interpreted as evidence of inappropriate or inferior care.

The PATONCOS tool enables the comparison of treatment costs across institutions using a standardized and clinically meaningful stratification system. By introducing the average cost per patient per month as a practical and reproducible economic indicator, this methodology facilitates the identification of variability in therapeutic strategies and supports benchmarking between centres. It should be noted that the current validation of this indicator is based on cost comparability; outcome-linked analyses will be necessary in future research to fully establish its value in clinical decision-making and resource allocation.

This approach contributes to a better understanding of the economic burden of cancer from the perspectives of patients, healthcare professionals, and health systems. Furthermore, it provides a practical tool for improving resource allocation and promoting more efficient and sustainable healthcare management.

The implementation of this methodology represents a step forward in aligning clinical decision-making with economic evaluation in oncology and hematology, particularly in the context of rapid therapeutic innovation.

## 6. Future Directions

Future research should focus on evaluating clinically meaningful outcomes, including overall survival, quality of life, end-of-life toxicity, and the use of chemotherapy in the last 30 days of life, stratified according to the current PATONCOS classification. This approach would enable a more comprehensive assessment of the relationship between healthcare resource utilization and patient outcomes.

The implementation of the PATONCOS tool across all healthcare facilities within a defined region would provide a detailed and structured overview of expenditure in one of the most economically impactful areas of healthcare systems. Prioritizing the most prevalent conditions and those associated with the highest economic burden may facilitate the identification of targeted strategies aimed at optimizing resource allocation and improving efficiency.

In this context, the PATONCOS framework has the potential to bridge the gap between the generation of high-quality scientific evidence, its translation into routine clinical practice, and its integration into healthcare budgeting processes. Expanding the use of this tool to additional centers, including national and international cohorts, would enhance the external validity of the classification system and improve the homogeneity of patient groups, allowing robust evaluation of its generalizability and scalability.

Furthermore, the incorporation of advanced information technologies, including artificial intelligence–based systems, could support automated patient classification and continuous updating of stratification criteria. The development of automated pre-assignment checklists and real-time alerts linked to changes in electronic health record data may reduce variability in patient classification and ensure dynamic reassessment throughout the clinical course.

Finally, the integration of clinical outcomes—such as overall survival, progression-free survival, and patient-reported outcomes—with economic data represents a key step toward value-based healthcare. Linking cost indicators with outcome measures would enable a more comprehensive evaluation of treatment effectiveness and support more informed, patient-centered decision-making in resource allocation. 

## Figures and Tables

**Table 1:** Representative PATONCO categories according to tumour type, therapeutic intent, and biomarker status.

Tumour Type	Representative PATONCO Categories
Breast cancer	Adjuvant HER2(+) HR(−); Adjuvant HER2(−) HR(+); Metastatic HER2(−) HR(+); Metastatic triple-negative
Colorectal cancer	Adjuvant colon cancer; Metastatic KRAS/NRAS mutated; Metastatic KRAS/NRAS wild-type
Lung cancer (NSCLC)	Adjuvant NSCLC; Metastatic non-squamous ALK(−)/EGFR(−); Metastatic ALK(+)/EGFR(+)
Ovarian cancer	Adjuvant ovarian cancer; Metastatic BRCA(+) first line; Platinum-resistant second line
Prostate cancer	Hormone-sensitive metastatic; Castration-resistant metastatic
Melanoma	Adjuvant melanoma; Metastatic BRAF(+); Metastatic BRAF(−)
Gastroesophageal cancer (GEJ)	Adjuvant adenocarcinoma; Metastatic HER2(+); Metastatic HER2(−)
Haematological malignancies	Hodgkin lymphoma; Large B-cell lymphoma; Multiple myeloma (transplant candidate)

**Table 2:** Average monthly cost per patient in the most representative PATONCO categories across participating hospitals.

PATONCO Category	H1	H2	H3	H4	Dispensed	Patients	H	*p*
MBC HER2(−) HR(+)	1546.76 (1198.78)	1192.50 (865.80)	1497.64 (1233.16)	1426.45 (861.11)	2079	285	21.63 **	0.000
MNSCLC ALK(−) EGFR(−)	1851.02 (1773.10)	2327.83 (1626.00)	1832.98 (1584.21)	1797.35 (1592.73)	1130	208	25.29 **	0.000
Adjuvant colon cancer	60.43 (61.49)	117.08 (568.66)	151.57 (546.73)	62.73 (32.37)	655	191	126.60 **	0.000
MCRC KRAS/NRAS mutated	972.40 (997.52)	791.77 (717.42)	502.62 (698.64)	542.36 (713.52)	999	175	94.04 **	0.000
MCRC KRAS/NRAS wild-type	1632.86 (1067.64)	1650.30 (1311.47)	1058.25 (1313.52)	1520.21 (1201.89)	1209	170	92.61 **	0.000
Castration-resistant metastatic prostate cancer	2387.42 (2030.45)	1915.45 (1944.07)	2962.25 (2228.91)	1780.68 (1311.32)	940	166	52.37 **	0.000
Multiple myeloma (transplant candidate)	2009.50 (2958.81)	1106.19 (2286.92)	2613.81 (3965.06)	3272.27 (4932.95)	1012	153	11.56 **	0.009
Adjuvant breast cancer HER2(−) HR(+)	238.04 (566.73)	41.90 (45.75)	465.41 (883.28)	182.58 (492.69)	457	117	71.04 **	0.000
Hormone-sensitive metastatic prostate cancer	2265.70 (2062.00)	2621.13 (1940.00)	2763.11 (2145.70)	1354.96 (971.46)	498	101	40.22 **	0.000
Metastatic small cell lung cancer	1163.57 (1296.99)	1283.81 (1243.63)	715.06 (875.21)	1253.02 (1272.92)	353	101	18.45 **	0.000

H1–H4: Hospital 1–4; MBC: metastatic breast cancer; MNSCLC: metastatic non-small cell lung cancer; MCRC: metastatic colorectal cancer. ** *p* < 0.01.

**Table 3:** Budget impact model based on average monthly cost per patient and number of patients across PATONCO categories.

PATONCO Category	H1	H2	H3	H4	Avg (€)	Dispensed	Patients	H	Impact (€)
MNSCLC ALK(−) EGFR(−)	1851.02	2327.83	1832.98	1797.35	1952.30	1130	208	25.29 **	406,077.36
MBC HER2(−) HR(+)	1546.76	1192.50	1497.64	1426.45	1415.84	2079	285	21.63 **	403,513.69
CR metastatic prostate cancer	2387.42	1915.45	2962.25	1780.68	2261.45	940	166	52.37 **	375,400.70
MM non-transplant	4002.05	2238.41	2219.96	2664.74	2781.29	815	112	61.33 **	311,504.48
MM transplant candidate	2009.50	1106.19	2613.81	3272.27	2009.50	1012	153	11.56 **	307,453.50
MNSCLC ALK(−) EGFR(+)	4507.12	3501.82	3749.08	4117.00	3968.76	424	75	8.35 *	297,656.63
MCRC KRAS/NRAS wt	1632.86	1650.30	1058.25	1520.21	1465.41	1209	170	92.61 **	249,118.85
HS metastatic prostate cancer	2265.70	2621.13	2763.11	1354.96	2251.23	498	101	40.22 **	227,373.73
NSCLC squamous	2055.38	2218.76	1532.76	1562.25	1842.29	392	97	20.71 **	178,701.89
Adjuvant NSCLC	1313.05	286.05	1045.29	2163.33	1507.22	377	113	8.06 *	170,316.24
MBC HER2(+) HR(+)	2685.88	2425.44	2495.37	2233.89	2460.15	625	65	7.19 NS	159,909.43
Neoadjuvant breast HER2(+)	2649.06	2208.15	1896.48	3266.54	2505.06	320	61	8.25 *	152,808.51
Neoadjuvant breast TN	2446.99	58.35	101.94	423.67	2446.99	295	62	141.47 **	151,713.38
Adjuvant breast HER2(+)	2143.24	1670.44	1766.15	1449.95	1757.45	510	80	28.42 **	140,595.60
Metastatic SCLC	1163.57	1283.81	715.06	1253.02	1233.47	353	101	18.45 **	124,580.13

H1–H4: Hospital 1–4; MBC: metastatic breast cancer; MNSCLC: metastatic non-small cell lung cancer; MCRC: metastatic colorectal cancer; HS: hormone-sensitive; CR: castration-resistant; MM: multiple myeloma. * *p* < 0.05; ** *p* < 0.01; NS: not significant.

## Data Availability

The data underlying this article are not publicly available due to patient privacy and institutional re-strictions. Aggregated anonymized data may be made available from the corresponding author upon reasonable request and with permission of Hospital Universitario Infanta Cristina.

## Supplementary Materials

The following supporting information can be downloaded at: https://www.mdpi.com/article/10.3390/jcm15114374/s1, Table S1: Patients classified according to PATONCOS system: Table S2: Average monthly cost by pathology category, broken down by hospital with standard deviation. Includes those whose sample of patients was greater than 50 per group; Table S3: Statistical analysis of the differences in cost/average/month/patonco category per hospital in top seven categories. Information in euros; Table S4: Budget calculation model based on average monthly consumption per patient and number of patients. Information on budget allocation for each category and weighted monthly economic weight. Information in euros.

## Abbreviations

The following abbreviations are used in this manuscript:

ACC	Adjuvant Colon Cancer
AJCC	American Joint Committee on Cancer
ASCO	American Society of Clinical Oncology
CRMPC	Castrate Resistant Metastatic Prostate Cancer
ESMO	European Society for Medical Oncology
ESMO-MCBS	European Society for Medical Oncology- Magnitude of Clinical Benefit Scale
GEJ	Gastric and gastroesophageal Junction
ICD-O	International Classification of Diseases for Oncology
MBC-RH	Metastatic Breast Cancer Postive Hormonal Receptor
MCC-KNM	Metastatic Colorectal Cancer Kras Nras Mutate
MCC-KNN	Metastatic Colorectal Cancer Kras Nras Native
MMTC	Multiple Myeloma Transplant Candidate
MNSCLC	Metastatic Non Small Cell Lung Cancer No Squamous
NSCLC	Non Small Cell Lung Cancer
RH	Hormonal Receptor
TDABC	Time Driven Activity-Based Costing
