# Supplementary material for: An Innovative Patient Stratification Tool Integrating Clinical and Economic Data for Benchmarking Oncology and Hematology Care: The PATONCOS System
Source: J Clin Med. 2026 Jun 5;15(11):4374. doi: 10.3390/jcm15114374 (PMC13257923; doi:10.3390/jcm15114374)
Supplement: Supplementary file 1 [file jcm-15-04374-s001.zip › ESMO (PATONCOS TOOL) Table S1.pdf]

Table S1. Patients classified according to PATONCOS system:

**PATONCO CATEGORY (I)**
HEAD AND NECK CANCER
METASTASIC HEAD AND NECK CANCER
ADJUVANT COLON CANCER
NEOADJUVANT COLON CANCER
METASTASIC COLORECTAL CANCER KRAS NRAS MUTA
METASTASIC COLORECTAL CANCER KRAS NRAS NATIV
METASTASIC COLORECTAL CANCER
ADJUVANT OESOPHAGEAL ADENOCARCINOMA
ADJUVANT SQUAMOUS OESOPHAGEAL CANCER
METASTASIC OESOPHAGEAL ADENOCARCINOMA
METASTASIC SQUAMOUS OESOPHAGEAL CANCER
NEOADJUVANT OESOPHAGEAL ADENOCARCINOMA
METASTASIC GEJ ADENOCARCINOMA HER2(-)
METASTASIC GEJ NO ADENOCARCINOMA HER2(-)
ADJUVANT GEJ ADENOCARCINOMA
ADJUVANT GEJ NO ADENOCARCINOMA
METASTASIC GEJ ADENOCARCINOMA HER2(+)
NEOADJUVANT GEJ ADENOCARCINOMA
ADJUVANT BREAST CANCER HER2(+) RH (-)
ADJUVANT BREAST CANCER HER2(+) RH (+)
ADJUVANT BREAST CANCER HER2(-) RH (+)
ADJUVANT BREAST CANCER TRIPLE-NEGATIVE
METASTASIC BREAST CANCER HER2(+) RH (-)
METASTASIC BREAST CANCER HER2(+) RH (+)
METASTASIC BREAST CANCER HER2(-) RH (+)
METASTASIC BREAST CANCER TRIPLE NEGATIVE
NEOADJUVANT BREAST CANCER HER2(+) RH (-)
NEOADJUVANT BREAST CANCER HER2(+) RH (+)
NEOADJUVANT BREAST CANCER HER2(-) RH (+)
NEOADJUVANT BREAST CANCER TRIPLE-NEGATIVE
ADJUVANT MELANOMA
METASTASIC MELANOMA BRAF(+)
METASTASIC MELANOMA BRAF(-)
ADJUVANT NSCLC
METASTASIC NSCLC SQUAMOUS ALK(-) EGFR (-)
METASTASIC NSCLC NO SQUAMOUS ALK(+) EGFR (+)
METASTASIC NSCLC NO SQUAMOUS ALK(+) EGFR (-)
METASTASIC NSCLC NO SQUAMOUS ALK(-) EGFR (+)
METASTASIC NSCLC NO SQUAMOUS ALK(-) EGFR (-)
ADJUVANT OVARIAN CANCER
METASTASIC OVARIAN CANCER BRCA(+) FIRST LINE
METASTASIC OVARIAN CANCER BRCA(-) FIRST LINE
METASTASIC OVARIAN CANCER BRCA(+) PLATINUM-RESISTANT SECOND LINE
**PATONCO CATEGORY (II)**

METASTASIC OVARIAN CANCER BRCA(-) PLATINUM-RESISTANT SECOND LINE
METASTASIC OVARIAN CANCER BRCA(+) PLATINUM-SENSITIVE SECOND LINE
METASTASIC OVARIAN CANCER BRCA(-) PLATINUM-SENSITIVE SECOND LINE
NEOADJUVANT OVARIAN CANCER
HORMONO-SENSITIVE METASTASIC PROSTATE CANCER
CASTRATE RESISTANT METASTASIC PROSTATE CANCER
PROSTATE CANCER NO METASTASIC
ADJUVANT RECTAL CANCER
NEOADJUVANT RECTAL CANCER
ADJUVANT SCLC
METASTASIC SCLC
HODGKIN LYMPHOMA
BURKITT LYMPHOMA
LARGE B-CELL LYMPHOMA
FOLLICULAR LYMPHOMA
T-CELL LYMPHOMA
MANTLE CELL LYMPHOMA
MULTIPLE MYELOMA TRANSPLANT CANDIDATE
MULTIPLE MYELOMA NON TRANSPLANT CANDIDATE

GEJ: Gastric and gastresophageal Junction; NSCLC: Non-Small Cell Lung Cancer; SCLC: Small Cell Lung Cancer; biomarkers considered: HER2, ALK, EGFR, BRAF, KRAS, NRAS, BRCA, RH
