# Supplementary material for: An Innovative Patient Stratification Tool Integrating Clinical and Economic Data for Benchmarking Oncology and Hematology Care: The PATONCOS System
Source: J Clin Med. 2026 Jun 5;15(11):4374. doi: 10.3390/jcm15114374 (PMC13257923; doi:10.3390/jcm15114374)
Supplement: Supplementary file 1 [file jcm-15-04374-s001.zip › ESMO (PATONCOS TOOL) Table S2.pdf]

TABLE S2:

Average monthly cost by pathology category, broken down by hospital with standard deviation. Includes those whose sample of patients was greater than 50 per group.

| PATONCO CATEGORY | Hospital 1 Average (Standard deviation) | Hospital 2 Average (Standard deviation) | Hospital 3 Average (Standard deviation) | Hospital 4 Average (Standard deviation) | Number dispensed lines | Number patients | H | p-value |
|---|---|---|---|---|---|---|---|---|
| **METASTASIC BREAST CANCER HER2(-) RH (+)** | 1546.76 (1198.78) | 1192.50 (865.80) | 1497.64 (1233.16) | 1426.45 (861.11) | 2079 | 285 | **21.63**** | **.000** |
| **METASTASIC NSCLC NO SQUAMOUS ALK(-) EGFR (-)** | 1851.02 (1773.10) | 2327.83 (1626.00) | 1832.98 (1584.21) | 1797.35 (1592.73) | 1130 | 208 | **25.29**** | **.000** |
| **ADJUVANT COLON CANCER** | 60.43 (61.49) | 117.08 (568.66) | 151.57 (546.73) | 62.73 (32.37) | 655 | 191 | **126.60**** | **.000** |
| **METASTASIC COLORECTAL CANCER KRAS NRAS MUTA** | 972.40 (997.52) | 791.77 (717.42) | 502.62 (698.64) | 542.36 (713.52) | 999 | 175 | **94.04**** | **.000** |
| **METASTASIC COLORECTAL CANCER KRAS NRAS NATIV** | 1632.86 (1067.64) | 1650.30 (1311.47) | 1058.25 (1313.52) | 1520.21 (1201.89) | 1209 | 170 | **92.61**** | **.000** |
| **CASTRATE RESISTANT METASTASIC PROSTATE CANCER** | 2387.42 (2030.45) | 1915.45 (1944.07) | 2962.25 (2228.91) | 1780.68 (1311.32) | 940 | 166 | **52.37**** | **.000** |
| **MULTIPLE MYELOMA TRANSPLANT CANDIDATE** | 2009.50 (2958.81) | 1106.19 (2286.92) | 2613.81 (3965.06) | 3272.27 (4932.95) | 1012 | 153 | **11.56**** | **.009** |
| **ADJUVANT BREAST CANCER HER2(-) RH (+)** | 238.04 (566.73) | 41.90 (45.75) | 465.41 (883.28) | 182.58 (492.69) | 457 | 117 | **71.04**** | **.000** |
| **ADJUVANT NSCLC** | 1313.05 (1834.84) | 286.05 (491.91) | 1045.29 (1610.10) | 2163.33 (2409.27) | 377 | 113 | **8.06*** | **.045** |
| **MULTIPLE MYELOMA NON TRANSPLANT CANDIDATE** | 4002.05 (3562.39) | 2238.41 (3105.28) | 2219.96 (3514.66) | 2664.74 (2851.86) | 815 | 112 | **61.33**** | **.000** |
| **HORMONO-SENSITIVE METASTASIC PROSTATE CANCER** | 2265.70 (2062.00) | 2621.13 (1940.00) | 2763.11 (2145.70) | 1354.96 (971.46) | 498 | 101 | **40.22**** | **.000** |
| **METASTASIC SCLC** | 1163.57 (1296.99) | 1283.81 (1243.63) | 715.06 (875.21) | 1253.02 (1272.92) | 353 | 101 | **18.45**** | **.000** |
| **HEAD AND NECK CANCER** | 1752.19 (1194.48) | 941.72 (1248.75) | 447.84 (941.82) | 719.13 (1281.10) | 298 | 98 | **58.35**** | **.000** |

TABLE S2 (continued):

Average monthly cost by pathology category, broken down by hospital with standard deviation. Includes those whose sample of patients was greater than 50 per category.

| PATONCO CATEGORY | **Hospital 1** Average (Standard deviation) | **Hospital 2** Average (Standard deviation) | **Hospital 3** Average (Standard deviation) | **Hospital 4** Average (Standard deviation) | Number dispensed lines | Number patients | H | p-value |
|---|---|---|---|---|---|---|---|---|
| **METASTASIC NSCLC SQUAMOUS ALK(-) EGFR (-)** | 2055.38 (1526.84) | 2218.76 (1377.45) | 1532.76 (1160.41) | 1562.25 (1503.12) | 392 | 97 | **20.71**** | **.000** |
| **METASTASIC COLORECTAL CANCER** | 532.93 (784.30) | - | 502.45 (929.57) | 311.47 (438.20) | 378 | 81 | 2.66[NS] | **.264** |
| **ADJUVANT BREAST CANCER HER2(+) RH (+)** | 2143.24 (1683.71) | 1670.44 (1427.67) | 1766.15 (1792.55) | 1449.95 (1872.64) | 510 | 80 | **28.42**** | **.000** |
| **FOLLICULAR LYMPHOMA** | 1170.24 (826.05) | 926.98 (467.26) | 823.29 (551.36) | 473.35 (201.11) | 306 | 80 | **28.42**** | **.000** |
| **METASTASIC NSCLC NO SQUAMOUS ALK(-) EGFR (+)** | 4507.12 (3212.43) | 3501.82 (1875.33) | 3749.08 (3279.18) | 4117.00 (1356.81) | 424 | 75 | **8.35*** | **.039** |
| **NEOADJUVANT BREAST CANCER HER2(-) RH (+)** | 209.15 (371.39) | 41.72 (21.48) | 262.73 (541.73) | 308.74 (641.95) | 353 | 74 | **37.83**** | **.000** |
| **METASTASIC BREAST CANCER HER2(+) RH (+)** | 2685.88 (1797.65) | 2425.44 (1831.92) | 2495.37 (1761.28) | 2233.89 (2269.29) | 625 | 65 | 7.19[NS] | **.066** |
| **LARGE B-CELL LYMPHOMA** | 716.41 (1137.88) | 1355.67 (1693.70) | 1159.09 (2628.03) | 563.86 (199.38) | 243 | 65 | **23.84**** | **.000** |
| **NEOADJUVANT BREAST CANCER TRIPLE-NEGATIVE** | 2446.99 (1371.31) | 58.35 (50.85) | 101.94 (245.38) | 423.67 (918.33) | 295 | 62 | **141.47**** | **.000** |
| **NEOADJUVANT BREAST CANCER HER2(+) RH (+)** | 2649.06 (1975.08) | 2208.15 (2087.55) | 1896.48 (1547.15) | 3266.54 (2315.44) | 320 | 61 | **8.25*** | **.041** |
| **METASTASIC OVARIAN CANCER BRCA(-) FIRST LINE** | 1105.81 (822.70) | 1466.90 (1736.49) | 1411.87 (1492.81) | 603.25 (799.06) | 305 | 59 | **9.31*** | **.025** |
| **METASTASIC GEJ ADENOCARCINOMA HER2(-)** | 1273.33 (1601.12) | 1661.85 (2916.10) | 589.67 (1169.55) | 191.45 (559.19) | 278 | 57 | **16.10**** | **.001** |
