# Supplementary material for: An Innovative Patient Stratification Tool Integrating Clinical and Economic Data for Benchmarking Oncology and Hematology Care: The PATONCOS System
Source: J Clin Med. 2026 Jun 5;15(11):4374. doi: 10.3390/jcm15114374 (PMC13257923; doi:10.3390/jcm15114374)
Supplement: Supplementary file 1 [file jcm-15-04374-s001.zip › ESMO (PATONCOS TOOL) Table S3.pdf]

TABLE S3: Statistical analysis of the differences in cost/average/month/patonco category per hospital in top seven categories. Information in euros

Patonco Category	H1 vs H2 Mean difference (p-value)	H1 vs H3 Mean difference (p-value)	H1 vs H4 Mean difference (p-value)	H2 vs H3 Mean difference (p-value)	H2 vs H4 Mean difference (p-value)	H3 vs H4 Mean difference (p-value)
ADJUVANT COLON CANCER	**-56.65****	**-91.14****	-2.30NS	-34.49NS	**54.36****	**88.84****
	**(.000)**	**(.000)**	(.212)	(.240)	**(.000)**	**(.000)**
METASTASIC COLORECTAL CANCER KRAS NRAS MUTA	180.62NS	**469.78****	**430.03****	**289.15****	**249.41****	-39.74NS
	(.052)	**(.000)**	**(.000)**	**(.000)**	**(.000)**	(.408)
METASTASIC COLORECTAL CANCER KRAS NRAS NATIV	-17.44NS	**574.61****	112.65NS	**592.05****	130.08NS	**-461.96****
	(.682)	**(.000)**	(.498)	**(.000)**	(.690)	**(.000)**
METASTASIC BREAST CANCER HER2(-) RH (+)	**354.25****	**49.12***	120.30NS	**-305.14***	**-233.95****	71.19NS
	**(.000)**	**(.016)**	(.443)	**(.017)**	**(.006)**	(.443)
METASTASIC NSCLC NO SQUAMOUS ALK(-) EGFR (-)	**-476.81****	18.03NS	53.67NS	**494.84****	530.47NS	35.63NS
	**(.002)**	(.581)	(.668)	**(.000)**	(.093)	(.581)
CASTRATE RESISTANT METASTASIC PROSTATE CANCER	471.98NS	**-574.82****	**606.74***	**-1046.80****	134.76NS	**1181.57****
	(.063)	**(.001)**	**(.041)**	**(.000)**	(.490)	**(.000)**
MULTIPLE MYELOMA TRANSPLANT CANDIDATE	**903.31***	-604.31NS	-1262.77NS	**-1507.62***	**-2166.08***	-658.46NS
	**(.026)**	-1.000	-1.000	**(.026)**	**(.028)**	(.986)
	*NS Not significant. * Significant. ** Very significant. H1: Hospital 1; H2: hospital 2; H3: hospital 3; H4: hospital 4*					
