# Supplementary material for: An Innovative Patient Stratification Tool Integrating Clinical and Economic Data for Benchmarking Oncology and Hematology Care: The PATONCOS System
Source: J Clin Med. 2026 Jun 5;15(11):4374. doi: 10.3390/jcm15114374 (PMC13257923; doi:10.3390/jcm15114374)
Supplement: Supplementary file 1 [file jcm-15-04374-s001.zip › ESMO (PATONCOS TOOL) Table S4.pdf]

TABLE S4: Budget calculation model based on average monthly consumption per patient and number of patients. Information on budget allocation for each category and weighted monthly economic weight. Information in euros

PATONCO CATEGORY	**Hospital 1** Average	**Hospital 2** Average	**Hospital 3** Average	**Hospital 4** Average	AVERAGE	Number dispensed lines	Number patients	H	p-value	Weighted monthly economic impact
**METASTASIC NSCLC NO SQUAMOUS ALK(-) EGFR (-)**	1851,02	2327,83	1832,98	1797,35	1.952,30 €	1130	208	**25.29****	**.000**	406.077,36 €
**METASTASIC BREAST CANCER HER2(-) RH (+)**	1546,76	1192,50	1497,64	1426,45	1.415,84 €	2079	285	**21.63****	**.000**	403.513,69 €
**CASTRATE RESISTANT METASTASIC PROSTATE CANCER**	2387,42	1915,45	2962,25	1780,68	2.261,45 €	940	166	**52.37****	**.000**	375.400,70 €
**MULTIPLE MYELOMA NON TRANSPLANT CANDIDATE**	4002,05	2238,41	2219,96	2664,74	2.781,29 €	815	112	**61.33****	**.000**	311.504,48 €
**MULTIPLE MYELOMA TRANSPLANT CANDIDATE**	2009,50	1106.19	2613.81	3272.27	2.009,50 €	1012	153	**11.56****	**.009**	307.453,50 €
**METASTASIC NSCLC NO SQUAMOUS ALK(-) EGFR (+)**	4507,12	3501,82	3749,08	4117,00	3.968,76 €	424	75	**8.35***	**.039**	297.656,63 €
**METASTASIC COLORECTAL CANCER KRAS NRAS NATIV**	1632,86	1650,30	1058,25	1520,21	1.465,41 €	1209	170	**92.61****	**.000**	249.118,85 €
**HORMONO-SENSITIVE METASTASIC PROSTATE CANCER**	2265,70	2621,13	2763,11	1354,96	2.251,23 €	498	101	**40.22****	**.000**	227.373,73 €
**METASTASIC NSCLC SQUAMOUS ALK(-) EGFR (-)**	2055,38	2218,76	1532,76	1562,25	1.842,29 €	392	97	**20.71****	**.000**	178.701,89 €
**ADJUVANT NSCLC**	1313,05	286,05	1045,29	2163,33	1.507,22 €	377	113	**8.06***	**.045**	170.316,24 €
**METASTASIC BREAST CANCER HER2(+) RH (+)**	2685,88	2425,44	2495,37	2233,89	2.460,15 €	625	65	7.19^{NS}	.066	159.909,43 €
**NEOADJUVANT BREAST CANCER HER2(+) RH (+)**	2649,06	2208,15	1896,48	3266,54	2.505,06 €	320	61	**8.25***	**.041**	152.808,51 €
**NEOADJUVANT BREAST CANCER TRIPLE-NEGATIVE**	2446,99	58,35	101,94	423,67	2.446,99 €	295	62	**141.47****	**.000**	151.713,38 €
**ADJUVANT BREAST CANCER HER2(+) RH (+)**	2143,24	1670,44	1766,15	1449,95	1.757,45 €	510	80	**28.42****	**.000**	140.595,60 €
**METASTASIC SCLC**	1163,57	1283,81	715,06	1253,02	1.233,47 €	353	101	**18.45****	**.000**	124.580,13 €

TABLE S4 (Continued) Budget calculation model based on average monthly consumption per patient and number of patients. Information on budget allocation for each category and weighted monthly economic weight. Information in euro

PATONCO CATEGORY	**Hospital 1** Average	**Hospital 2** Average	**Hospital 3** Average	**Hospital 4** Average	**AVERAGE**	Number dispensed lines	Number patients	**H**	**p-value**	Weighted monthly economic impact
**METASTASIC COLORECTAL CANCER KRAS NRAS MUTA**	972,40	791,77	502,62	542,36	702,29 €	999	175	**94.04****	**.000**	122.900,31 €
**HEAD AND NECK CANCER**	1752,19	941,72	447,84	719,13	1.137,68 €	298	98	**58.35****	**.000**	111.492,64 €
**FOLLICULAR LYMPHOMA**	1170,24	926,98	823,29	473,35	1.170,24 €	306	80	**28.42****	**.000**	93.619,20 €
**METASTASIC OVARIAN CANCER BRCA(-) FIRST LINE**	1105,81	1466,90	1411,87	603,25	1.328,19 €	305	59	**9.31***	**.025**	78.363,41 €
**METASTASIC BREAST CANCER HER2(+) RH (-)**	3146,68	1171,67	3387,70	2098,40	2.451,11 €	264	31	**33.03****	**.000**	75.984,49 €
**LARGE B-CELL LYMPHOMA**	716,41	1355,67	1159,09	563,86	1.077,06 €	243	65	**23.84****	**.000**	70.008,68 €
**METASTASIC GEJ ADENOCARCINOMA HER2(-)**	1273,33	1661,85	589,67	191,45	1.174,95 €	278	57	**16.10****	**.001**	66.972,15 €
**METASTASIC COLORECTAL CANCER**	832,93	-	502,45	311,47	548,95 €	378	81	2.66[NS]	.264	44.464,95 €
**ADJUVANT BREAST CANCER HER2(-) RH (+)**	238,04	41,90	465,41	182,58	231,98 €	457	117	**71.04****	**.000**	27.141,95 €
**ADJUVANT COLON CANCER**	60,43	117,08	151,57	62,73	97,95 €	655	191	**126.60****	**.000**	18.708,93 €
**NEOADJUVANT BREAST CANCER HER2(-) RH (+)**	209,15	41,72	262,73	308,74	205,59 €	353	74	**37.83****	**.000**	15.213,29 €
**NEOADJUVANT RECTAL CANCER**	118,27	46,72	37,89	87,06	72,49 €	91	31	**9.90***	**.019**	2.247,04 €
